# A Rare Case of Pontocerebellar Hypoplasia Type 1B With Literature Review

**DOI:** 10.7759/cureus.27098

**Published:** 2022-07-21

**Authors:** Ana C Spyridakis, Ying Cao, Florentina Litra

**Affiliations:** 1 Department of Child Neurology, St. Louis University, St. Louis, USA; 2 Pediatrics, University of Florida, Pensacola, USA; 3 Department of Pediatrics, Ascension Sacred Heart, Pensacola, USA

**Keywords:** pediatric genetics, genetic testing, pontocerebellar hypoplasia, genetic disorder, neonatal hypotonia

## Abstract

A full-term female newborn was transferred to our neonatal intensive care unit (NICU) on day two of life for hypotonia. Physical examination was significant for overriding sutures, displaced small anterior fontanelle, axial hypotonia, extremity hypertonia, and slow deep tendon reflexes. She was also noted to have stridor with crying but had unlabored breathing without oxygen requirements and a normal heart examination. A brain magnetic resonance imaging (MRI) showed a large cisterna magna and cerebellar hypoplasia with the majority of the cerebellar vermis present, suggesting a possible Dandy-Walker variant (cerebellar vermis hypoplasia). Head computed tomography showed areas of close approximation of coronal sutures and no synostosis. During the NICU stay, our patient was evaluated by Pediatric Neurology who recommended a chromosomal microarray which returned normal. The patient also had some difficulty feeding initially, but she was able to feed efficiently and gain weight by the time of discharge.

After discharge from NICU, her neurological status steadily declined, resulting in poor motor function and poor suck despite regular physical therapy, occupational therapy, and speech therapy. By three months of age, she developed failure to thrive and was admitted to the hospital for evaluation of the cause. Her neurological examination showed worsening of her axial hypotonia with very little movement in the upper extremities and hypertonia in the lower extremities. She had a weak suck with the inability to form a good seal on the nipple. A new heart murmur was noted and an echocardiogram showed a moderate-to-large atrial septal defect. A modified barium swallow study showed severe dysphagia for which she required gastrostomy tube placement for feeding. At follow-up with Neurology, she was noted to have progressive microcephaly, profound hypotonia, areflexia, and nystagmus. A second MRI showed worsening atrophy and increasing ventriculomegaly. By nine months of age, she developed respiratory failure, required a tracheostomy, and remained ventilator-dependent.

Genetics was then consulted and recommended a brain malformation genetic panel. The patient was found to be heterozygous for two pathogenic variants in the *EXOSC3* gene: c.155delC and D132A, which is consistent with a diagnosis of autosomal recessive pontocerebellar hypoplasia (PCH) type 1B. The mother was found to be a heterozygous carrier of the c.155delC pathogenic variant, while the father was a heterozygous carrier for the D132A variant, which confirms that the two variants are present on opposite alleles.

PCH describes a rare group of 11 neurodegenerative disorders that are typically seen prenatally or shortly after birth. PCH1 is characterized as a combination of PCH and spinal muscular atrophy, with patients presenting with muscle weakness and global developmental delay. An increased understanding of PCH1 will lead to better care and counseling for patients and families.

## Introduction

Pontocerebellar hypoplasia (PCH) comprises a clinically and genetically heterogeneous group of very rare disorders characterized mainly by hypoplasia and degeneration of the pons and cerebellum [1]. Patients presented with hypoplasia or atrophy of the cerebellum and pons, with variable involvement of supratentorial structures and motor and cognitive impairments [2]. The exact incidence of PCH is unknown due to its rarity. PCH1 is characterized by anterior horn degeneration in the spinal cord with muscle weakness and hypotonia. Here, we present a case of PCH type 1B with *EXOSC3 *gene mutation along with a literature review.

This article was previously presented as a meeting poster at the 2021 Virtual Southern Regional Meeting on February 25, 2021.

## Case presentation

A two-day-old full-term Caucasian female newborn was transferred to our neonatal intensive care unit for hypotonia. She was born at 38 weeks via C-section due to non-reassuring fetal heart tones. Apgar score was 5/8 at one minute and five minutes. Amniotic fluid was clear. All maternal serologies were negative, except Rubella equivocal. Prenatal history was pertinent for advanced maternal age (36 years), two early miscarriages, in vitro fertilization, and an adequate follow-up with high-risk maternal-fetal medicine. The patient had an unremarkable prenatal course, including normal prenatal ultrasounds, except for oligohydramnios developed at 36 weeks gestation. The patient’s family history was negative for neurological disorders. Neonatal intensive care unit (NICU) referred to developmental pediatrics, physical therapy, occupational therapy, and speech therapy with neurology follow-up for a presumed diagnosis of the Dandy-Walker variant based on the brain magnetic resonance imaging (MRI) findings.

After discharge, her neurological status steadily declined despite regular therapies. By three months of age, she developed failure to thrive due to ineffective suck, requiring gastrostomy tube placement for feeding. At six months of age, she was noted to have progressive microcephaly, profound hypotonia, areflexia, and nystagmus with worsening brain MRI appearance. By eight months of age, she developed respiratory failure secondary to respiratory muscle weakness, required a tracheostomy, and remained ventilator-dependent.

At two days of age, the physical examination was significant for stridor with crying, otherwise, unlabored breathing. At three months, she was noted to have very little movement in the upper extremities, hypertonia in the lower extremities, and a poor suck. At six months, she had progressive microcephaly, profound hypotonia, areflexia, and nystagmus.

**Figure 1:**
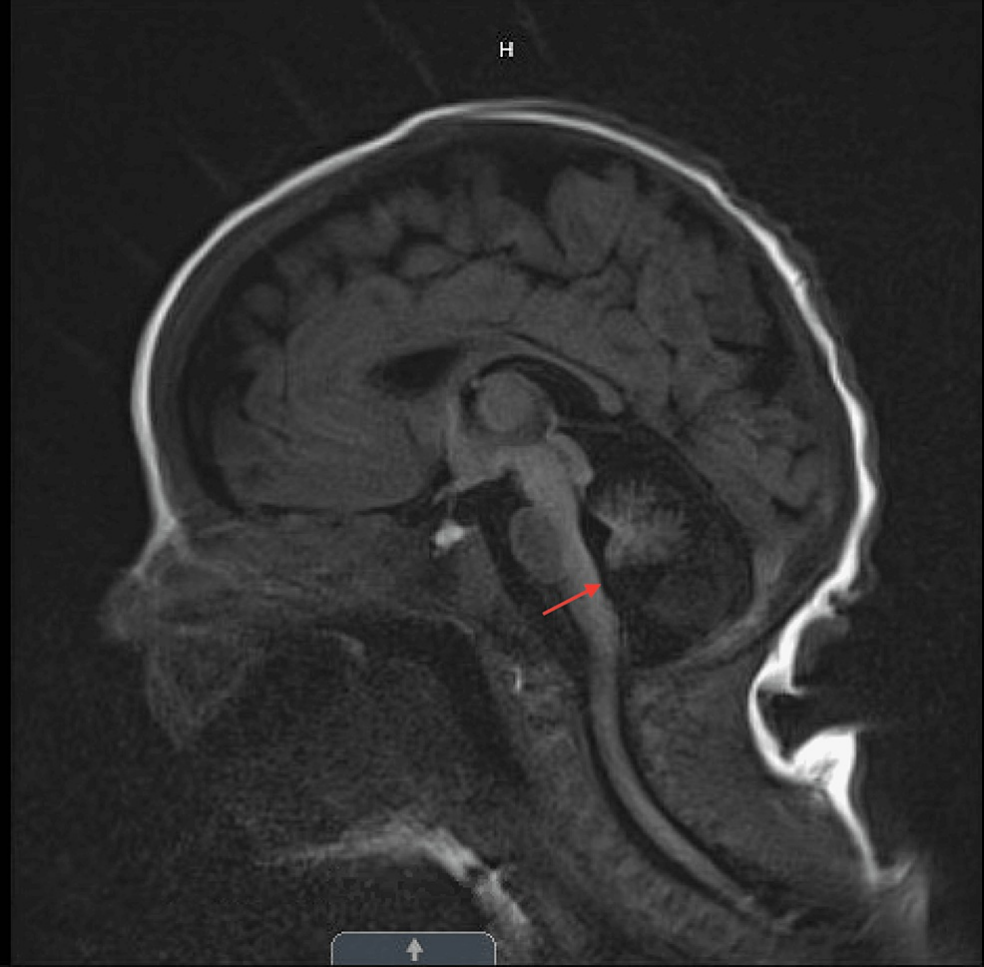
T1-blade sag on day two of life. Large cisterna magna and cerebellar hypoplasia with the majority of the cerebellar vermis can be seen, suggesting a possible Dandy-Walker variant (cerebellar vermis hypoplasia).

**Figure 2:**
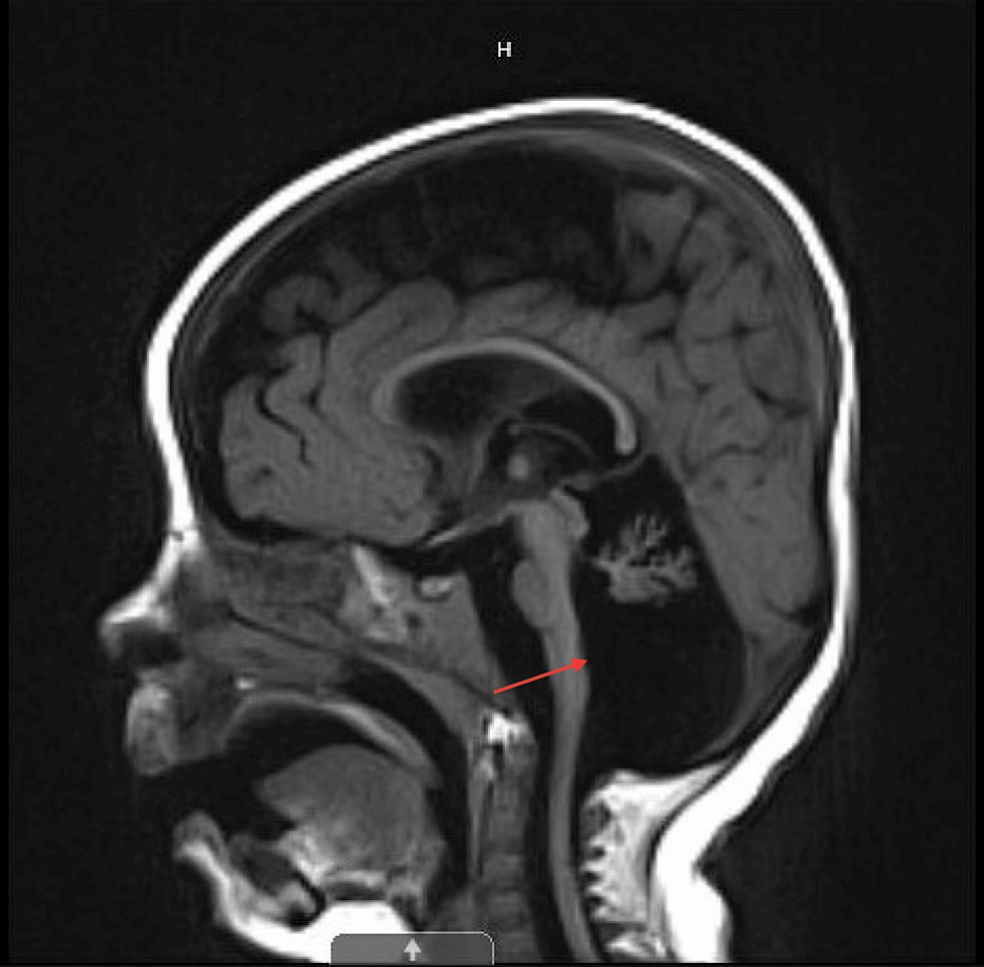
T1-blade sag at six months of life. Stable global white matter loss, diminished size of the brainstem, and profound decrease in the cerebellum can be seen with cerebellar disruption, pontocerebellar hypoplasia, and global cerebellar hypoplasia.

**Figure 3:**
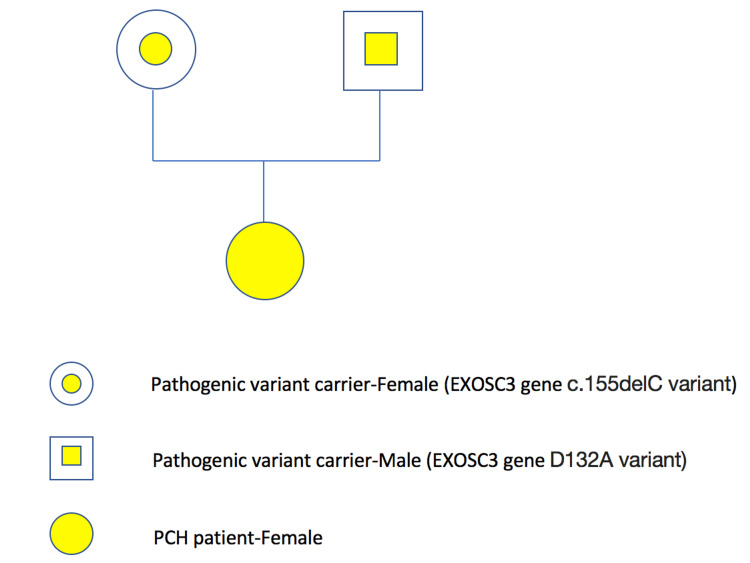
The genogram for the PCH case. PCH: pontocerebellar hypoplasia

During her NICU stay, the brain MRI showed a large cisterna magna and cerebellar hypoplasia with the majority of the cerebellar vermis present, suggesting a possible Dandy-Walker variant (cerebellar vermis hypoplasia) (Figure 1). Pediatric Neurology recommended chromosomal microarray which was normal. At three months, an echocardiogram was performed due to a new systolic murmur and showed a moderate-to-large atrial septal defect with bilateral atrial dilation, but normal ventricular function. At eight months, a repeat brain MRI showed stable global white matter loss, diminished size of the brainstem, and a profound decrease in the cerebellum that can be seen with cerebellar disruption, PCH, and global cerebellar hypoplasia (Figure 2). Genetics recommended neurologic gene testing, which resulted in three variants in the *SGCA*, *TTN*, and *COL6A2* genes, which can be seen with muscular dystrophies, but did not fully explain our patient’s symptoms. Further investigation with the Brain Malformation Genetic panel was suggested. The patient was found to be heterozygous for two pathogenic variants in the *EXOSC3 *gene: heterozygous c.155delC, p.P52RfsX2 and heterozygous c.395 A>C, p.D132A, which is consistent with a diagnosis of autosomal recessive PCH type 1B. Both parents underwent genetic investigations. The mother was found to be a heterozygous carrier of the c.155delC pathogenic variant, while the father was a heterozygous carrier for the D132A variant, which confirms that the two variants are present on opposite alleles (Figure 3).

## Discussion

Definition and classification

PCH comprises a clinically and genetically heterogeneous group of very rare disorders characterized mainly by hypoplasia and degeneration of the pons and cerebellum [1]. The classification of PCH was initially based on distinct clinical, radiological, and biochemical features (such as optic atrophy and cerebrospinal fluid lactate elevation). However, it is now mainly based on distinct clinical features and associated genetic defects. The current classification comprises 11 types of PCH. All PCH subtypes are inherited in an autosomal recessive manner.

PCH refers to a group of 11 different subtypes of phenotypes and clinical presentations. The different subtypes are characterized by dysfunction of the cortex and basal ganglia, leading to poor developmental progression and often early lethality [1,2]. PCH1 is characterized by PCH with bulbar and spinal motor neurodegeneration, which is also seen in spinal muscular atrophy (SMA). Currently, four genes are associated with PCH1 (*PCH1A-D*) [2].

*EXOSC3 *mutations have been identified in approximately half of the patients with PCH1. The *EXOSC3 *gene encodes component three of the RNA exosome complex, which is involved in mRNA degradation. One study found that mutations in *EXOSC3 *were clinically associated with prolonged survival (mean age at death was nine months in patients with mutations versus three months in patients without *EXOSC3 *mutations) [1]. The presentation of *EXOSC3*-mediated PCH1 mutation can further be differentiated into mild, moderate, and severe with various genotypes. There is a clear genotype-phenotype correlation

Patients with a homozygous c.395A>C, p.D132A mutation have a prolonged disease course with possible survival into early puberty and preservation of the pons compared to patients with a p.D132A allele plus a nonsense or p.Y109N mutation. Patients with a homozygous p.G31A mutation and those with a homozygous p.G135E mutation present a similarly severe phenotype with death in infancy [3].

Patients with homozygous missense mutations c.92G>C or c.395A>C in combination with deletion mutations, for example, c.155delC, p.52fs or c.551delG, p.184fs, are associated with the severe phenotype [1]. Our patient presented combined c.395 A>C, p.D132A with c155delC, p.P52RfsX2 mutations which have been found to be related to the severe phenotype described above.

The *EXOSC8 *gene also encodes an essential component of the RNA exosome complex. *EXOSC8 *mutations were found to cause psychomotor retardation, severe muscle weakness, spasticity, hearing and vision impairment, and motor neuron degeneration in 22 patients from three families. Neuroimaging showed hypoplasia of the cerebellum vermis and corpus callosum, cortical atrophy, and immature myelination [4].

Mutation in *SLC24A46 *was found to be associated with lethal PCH. This mutation was also found to be the underlying genetic cause in the original Dutch PCH1 family [5].

*VRK1 *mutations were described in one consanguineous PCH1 family of Ashkenazi Jewish origin which is a very rare cause of PCH1 [6]. *VRK1 *mutations were later found to be predominantly associated with motor neuron disease without PCH [2].

The cerebellum begins to differentiate in the fourth week of gestation but extends to about 20 months postnatally to mature. This may explain why some patients with PCH1 display normal brain structure prenatally by imaging methods, followed by rapidly losing the cerebellar hemisphere by weeks to months. As such, PCH is characterized by neuronal loss in the ventral pons and cerebellum, with onset in utero but continuing after birth [7].

The most important differential diagnosis of PCH1 is infantile SMA, which is caused by mutations in the *SMN1 *gene. In patients with PCH1, no mutations in *SMN1 *have yet been found; hence, PCH1 appears to be a distinct entity despite the shared involvement of spinal motor neurons [1].

Once the diagnosis has been established, it is important to provide counseling to families regarding recurrence risk and discuss reproductive options. Detection of PCH via ultrasound is unreliable as it fails to detect structural cerebral anomalies at routine screenings done at 20 weeks [1]. In families where a specific gene defect has been identified, particularly in families that underwent in vitro fertilization, it is important to offer a preimplantation genetic diagnosis or invasive prenatal testing [1].

Clinical presentation

Patients with PCH have generalized hypotonia, progressive muscular atrophy, and global developmental delay. In the case of our patient, PCH1 was characterized by the typical aforementioned PCH symptoms with the addition of bulbar and spinal motor degeneration which is also seen in SMA. Commonly seen clinical features include severe hypotonia, mostly absent motor or speech development, oculomotor dysfunction, and a lack of fixation. Patients are characterized to have progressive microcephaly, with normal birth measurements. In addition, there is increased spasticity, increased tendon reflexes, and decreased muscle tone along with dystonic movements.

Patients with *pD132A/EXOSC3* mutation, like in our case, tend to have a combination of upper and lower motor neuron signs. They may also exhibit dystonia and dyskinesia. Ataxia is notably absent in this specific mutation. There can be a delay in motor and non-motor development with variable severity.

Diagnosis

Currently, there are four gene mutations that have been associated with PCH1, one of which is *EXOSC3*, found in our patient [1]. In some patients, cerebellar hypoplasia is variably present and the pons may be unaffected depending on the severity of the symptoms. In mild cases, there can be hypoplasia of the cerebellar hemispheres with preserved folia and small, structurally intact pons. In more severe cases, there can be a significant reduction of the cerebellum, pons, and brainstem that is progressive. 

Diagnosis can be made prenatally for PCH1. Patients with PCH1 can have an SMA type 1 clinical presentation as it comprises progressive cerebellar and brainstem lesions with degeneration of motor neurons in the region of the anterior spinal horn. PCH1 can present with many different phenotypes, including various combinations of lower and upper motor neuron signs, ataxia, visual and hearing impairment, seizures, microcephaly, global developmental delay, nystagmus, and optic atrophy. More than half of PCH1 cases are caused by *EXOSC3*, which encodes subunit 3 of the human exosome complex, a major RNA processor in cells.

Our patient’s first genetic test resulted in three variants in the *SGCA*, *TTN*, and *COL6A2* genes, which did not explain her symptoms. The variant in *SGCA *was not suspicious. *SGCA *is associated with an autosomal recessive limb-girdle muscular dystrophy. The *TTN *variant is associated with an autosomal dominant form of cardiomyopathy; however, this myopathy usually presents later in life and is not associated with any issues in infancy. *COL6A2 *variant is associated with Ullrich congenital muscular dystrophy and Bethlem myopathy. The course is characterized by a progressive decline in motor and respiratory function in the first decade of life, with a majority confined to wheelchairs by 11 years of age [8]. However, because this variant is frequently seen in the normal population, it is relatively common without any associated pathogenic effects. Our geneticist doubted these findings explained our patient’s symptoms, and a brain malformation panel was ordered which revealed PCH1-related gene variation.

Prognosis and patient outcomes

In the neonatal period, neurological signs are commonly present. It has been suggested that there is no correlation between age at neurological onset and duration of life. This may indicate that the disease course is much more complex than originally thought and that the rate and course of the disease are variable for each mutation group. It is thought that the severity of motor delay is not correlated directly with the duration of life, but it is possible that it is correlated with the rate of progression of respiratory muscle weakness.

Patients who have null mutations (such as frameshift mutations) will have more severe presentations in heterozygous states and can be lethal in the homozygous state. Patients with a missense mutation tend to have a more variable presentation. Interestingly, even among similar genetic mutations, there is great variability in presentation as well as lifespan. The outcome of this disease ranges from neonatal death to survival into puberty. In the case of *EXOSC3* mutation in conjunction with homozygous c.395A>C, p.D132A mutations, patients tend to have a more mild course and can survive into early adulthood.

However, in patients who showed a more moderate disease course, mutations seen with the c.395A>C in combination with an insertion mutation c.226dupG, splice mutation at c.475-12A>G, or a disruption of the start codon c.2T>C would result in lifespans between one and three years. Lastly, patients with the more severe form, such as our patient, typically only survive up until the first year of life. These patients could exhibit missense mutations distinct from c.395A>C or with c.395A>C in combination with other deletion mutations. These patients typically require respiratory support very early in their course [1]. Our patient was discharged home with hospice care and expired at 14 months of age due to complications from respiratory failure.

## Conclusions

This case highlights the challenges associated with diagnosing rare neurological disorders in early childhood and providing adequate counseling to parents. This patient was initially diagnosed with a Dandy-Walker variant and thought to have a relatively good prognosis with intensive therapy. It was only after significant loss of milestones and neurological deterioration noted on serial examinations that we started questioning the diagnosis. The initial genetic results can be misleading or ambiguous, like in this case, and may give parents false expectations. Having a definitive diagnosis linked to an actual prognosis, however dismal, can give parents solace and time to prepare for the inevitable.

In this case, an interesting observation was the maternal history of two early miscarriages which may suggest a genetic component. In addition, our patient was conceived via in vitro fertilization and one may wonder about the value of preimplantation genetic testing. However, given that targeted genetic analysis is required for diagnosis, it is likely that the prenatal diagnosis of PCH would have been missed.
