# Correction: A Rare Case of Pontocerebellar Hypoplasia Type 1B With Literature Review

**DOI:** 10.7759/cureus.c100

**Published:** 2023-02-09

**Authors:** Ana C Spyridakis, Ying Cao, Florentina Litra

**Affiliations:** 1 Department of Child Neurology, St. Louis University, St. Louis, USA; 2 Pediatrics, University of Florida, Pensacola, USA; 3 Department of Pediatrics, Ascension Sacred Heart, Pensacola, USA

This article has been corrected at the request of the authors to change the following:

The affiliation of Ana C. Spyridakis has been changed from Washington University School of Medicine in St. Louis to St. Louis University.

The authors deeply regret that these errors were not identified and addressed prior to publication.

 

